# Multidrug-Resistance Patterns and Predictors in Adult Acute Pyelonephritis: A Three-Year Cohort from a Tertiary Romanian Center with Derivation of the PYELO-MDR-Risk Score

**DOI:** 10.3390/biomedicines14061264

**Published:** 2026-06-01

**Authors:** Livia Stanga, Ovidiu Rosca, Iulia Georgiana Bogdan, Ciprian Ilie Roșca, Horia Silviu Branea, Camelia Vidița Gurban, Marius Papurica

**Affiliations:** 1 Discipline of Microbiology, Faculty of Medicine, Victor Babes University of Medicine and Pharmacy, 300041 Timisoara, Romania; stanga.livia@umft.ro; 2 Department of Infectious Diseases, Victor Babes University of Medicine and Pharmacy, 300041 Timisoara, Romania; rosca.ovidiu@umft.ro (O.R.); iulia-georgiana.bogdan@umft.ro (I.G.B.); 3 Department V, Internal Medicine I—Discipline of Medical Semiology I, Center of Advanced Research in Cardiology and Hemostaseology, Victor Babes University of Medicine and Pharmacy, 300041 Timisoara, Romania; 4 Department of Internal Medicine I—Medical Semiotics II, Victor Babes University of Medicine and Pharmacy, 300041 Timisoara, Romania; 5 Department IV: Biochemistry and Pharmacology, Discipline of Biochemistry, Victor Babes University of Medicine and Pharmacy, Eftimie Murgu Square 2, 300041 Timisoara, Romania; gurban.camelia@umft.ro; 6 Anaesthesia and Intensive Care Research Center, Faculty of Medicine, Victor Babes University of Medicine and Pharmacy, Eftimie Murgu Square 2, 300041 Timisoara, Romania; papurica.marius@umft.ro

**Keywords:** pyelonephritis, drug resistance, multiple, bacterial, anti-bacterial agents, *Enterobacteriaceae* infections, risk assessment

## Abstract

**Background and Objectives**: Multidrug-resistant (MDR) uropathogens are reshaping the empirical management of acute pyelonephritis, particularly in Eastern European centers. We aimed to describe MDR patterns, identify admission-level predictors, including renal impairment/renal-failure status at presentation and major healthcare exposure variables, and derive a bedside risk score (PYELO-MDR-Risk) for adult pyelonephritis at a Romanian tertiary hospital. **Methods**: We retrospectively analyzed 129 consecutive culture-confirmed acute pyelonephritis admissions at “Victor Babeș” University Hospital, Timișoara (March 2022–March 2025). MDR was defined as non-susceptibility to ≥1 agent in ≥3 antimicrobial categories. We compared MDR and non-MDR cases on demographics, microbiology, time-to-effective therapy (TTE), and outcomes; multivariable logistic regression identified independent predictors and was the basis for a points-based score with bootstrap-based internal validation (1000 resamples). **Results**: Fifty-four patients (41.9%) had MDR pyelonephritis. *Escherichia coli* remained the dominant uropathogen (55.8%) but was less prevalent in the MDR group (40.7% vs. 66.7%; *p* = 0.003), whereas *Klebsiella pneumoniae* and *Pseudomonas aeruginosa* were enriched. Independent predictors of MDR were antibiotic exposure ≤90 days (aOR 5.7, 95% CI 2.4–13.6), recurrent UTI (aOR 3.4, 1.4–8.2), recent hospitalization (aOR 3.1, 1.2–8.0), and renal impairment/renal-failure status at admission (aOR 2.4, 1.0–6.2). Immunosuppression, prior urinary tract instrumentation, and nephrolithiasis/urolithiasis were evaluated as candidate predictors but did not independently improve the final point score after adjustment. MDR was associated with delayed effective therapy (28.4 vs. 9.7 h; *p* < 0.001), longer hospitalization (13.7 vs. 8.9 days; *p* < 0.001), and higher 30-day readmission (20.4% vs. 8.0%; *p* = 0.038). The PYELO-MDR-Risk score (range 0–12) achieved an optimism-corrected AUC of 0.84 with adequate calibration (Hosmer–Lemeshow *p* = 0.624). **Conclusions**: MDR drives a substantial fraction of pyelonephritis admissions in Western Romania and tracks closely with prior antibiotic and healthcare exposure. The PYELO-MDR-Risk score offers a transparent bedside tool for empirical-therapy decisions in the local setting, pending national and international external validation.

## 1. Introduction

Acute pyelonephritis remains one of the most common infectious admissions to internal medicine and infectious-disease wards across Europe, accounting for a substantial fraction of unscheduled antimicrobial prescribing and hospital occupancy [1,2]. Although the syndrome is broadly recognizable, its clinical heterogeneity—from young women with uncomplicated infection to elderly polymorbid patients with urological obstruction—translates into widely varying microbiology, therapeutic adequacy, and outcomes [2]. In recent years, the increasing prevalence of multidrug-resistant (MDR) uropathogens has further complicated empirical decision-making, especially in centers serving older populations with frequent healthcare contact [1,3].

Romania consistently reports some of the highest rates of antimicrobial resistance in the European Union. Surveillance data from the European Centre for Disease Prevention and Control (ECDC) place Romania among the leading countries for resistance among *Enterobacterales*, including extended-spectrum β-lactamase (ESBL) production and carbapenem non-susceptibility [3]. These patterns are most pronounced in tertiary hospitals concentrated in larger urban areas such as Timișoara, where referral pathways gather complex cases, surgical complications, and patients with recurrent antibiotic exposure. The interface between community-onset and healthcare-associated pyelonephritis is, therefore, particularly fluid in this setting and warrants explicit examination [3,4,5].

Several patient-level risk factors for MDR uropathogens have been described, including prior antibiotic use, recent hospitalization, indwelling urinary devices, nephrolithiasis/urolithiasis, diabetes mellitus, renal impairment, immunosuppression, and prior urinary tract instrumentation [4,5]. These variables are clinically important because they capture different mechanisms of resistance risk: antibiotic selection pressure, healthcare-associated colonization, impaired host defense, urinary stasis or obstruction, and procedure-related introduction of resistant flora. However, most published predictive models have been developed in Western European or North American populations, where baseline resistance rates differ substantially from those observed in South-Eastern Europe [4,6,7,8,9]. The transferability of such models to Romanian clinical practice has not been systematically evaluated, and locally calibrated tools are scarce. This gap is particularly relevant for inpatient pyelonephritis, where the empirical regimen chosen at admission is often the single strongest determinant of subsequent clinical trajectory [5,10,11].

Beyond the simple presence of MDR organisms, growing attention is being paid to the architecture of resistance—the way in which different resistance phenotypes co-occur within a single isolate [6]. Co-resistance patterns shape both the probability that a given empirical regimen will be active and the selection pressure exerted by re-treatment failures. Analyses based on Jaccard similarity and pairwise odds ratios have begun to map these clusters in *Enterobacterales* globally, identifying particularly tight links between ESBL production, third-generation cephalosporin resistance, and fluoroquinolone resistance [6,7]. Reproducing such analyses in local cohorts can directly inform empirical-therapy escalation rules and stewardship feedback to prescribers [7,10].

In addition to microbiological characterization, clinical decision support increasingly relies on structured risk scores that integrate readily available bedside information [8,9]. Although several scores exist for community-acquired UTI and complicated UTI in the outpatient setting, few have been specifically derived for hospitalized acute pyelonephritis with MDR as the primary endpoint [9,11]. A pragmatic, transparent, points-based score with internal validation could complement local antibiograms by helping clinicians stratify patients at admission and prioritize broad-spectrum empirical coverage for those most likely to harbor MDR pathogens, while sparing low-risk patients from unnecessary broad-spectrum exposure [8,10,11,12,13,14].

Against this background, the present study had three a priori objectives. First, we aimed to describe the MDR landscape and pathogen distribution among adults hospitalized with acute pyelonephritis at a tertiary university hospital in Western Romania between March 2022 and March 2025 [3,15]. Second, we sought to identify independent clinical predictors of MDR using multivariable logistic regression, with particular attention to modifiable exposures, such as prior antibiotic use, hospitalization, and urinary catheterization/instrumentation, as well as immunosuppression and nephrolithiasis/urolithiasis [4,5]. Third, we derived and internally validated a bedside risk score—PYELO-MDR-Risk—and explored its calibration, decision-relevant cut-points, and behavior across clinically meaningful subgroups, framing the analysis within the broader context of antimicrobial stewardship in Eastern European pyelonephritis care [9,10].

## 2. Materials and Methods

### 2.1. Study Design and Setting

This was a single-center, retrospective observational cohort study conducted at the “Victor Babeș” Clinical Hospital for Infectious Diseases and Pneumology, affiliated with the “Victor Babeș” University of Medicine and Pharmacy of Timișoara, Romania. The hospital functions as a tertiary referral center for infectious diseases and serves the Banat region in Western Romania, with an annual catchment of approximately 1.9 million inhabitants. The infectious-disease wards admit both community-onset and hospital-referred adults with suspected systemic infections, including acute pyelonephritis. Care is delivered by an interdisciplinary team comprising infectious-disease specialists, internists, microbiologists, and pharmacists.

Consecutive adult patients (≥18 years) admitted between 1 March 2022 and 31 March 2025 with a discharge diagnosis of acute pyelonephritis (ICD-10) and a microbiologically confirmed urinary pathogen were considered for inclusion. The study period was selected to cover three full years of post-pandemic clinical activity and to encompass the implementation of an updated institutional antibiogram in late 2022. Data were extracted from the hospital electronic medical record, the laboratory information system, and pharmacy dispensing records. The study was conducted in accordance with the Declaration of Helsinki and approved by the local research ethics committee; given the retrospective design and use of de-identified routinely collected data, the requirement for individual informed consent was waived.

### 2.2. Inclusion Criteria, Definitions, and MDR Classification

Eligible patients fulfilled all of the following criteria: (i) age ≥ 18 years; (ii) clinical syndrome compatible with acute pyelonephritis, defined as the combination of fever (≥38.0 °C) or chills with at least one of flank pain, costovertebral angle tenderness, or nausea/vomiting, accompanied by pyuria on microscopic urinalysis (≥10 leukocytes per high-power field) [1,2]; (iii) a positive urine culture obtained before antibiotic administration or within 24 h of admission, with growth of ≥10^5^ colony-forming units (CFU)/mL of a single uropathogen, or ≥10^4^ CFU/mL in patients with concordant blood cultures; and (iv) initiation of intravenous antibiotic therapy during the index admission. Pregnant patients, those with isolated lower urinary tract infection [11], perinephric abscess requiring drainage, or polymicrobial cultures with more than two organisms were excluded.

Multidrug resistance was defined according to the 2012 ECDC/CDC consensus as non-susceptibility (intermediate or resistant) to ≥1 agent in ≥3 antimicrobial categories relevant to the species in question [4]. Susceptibility testing was performed using the Vitek 2 system (bioMérieux, Marcy l’Étoile, France) and interpreted according to the European Committee on Antimicrobial Susceptibility Testing (EUCAST) breakpoints valid at the time of isolation [12]. ESBL production was confirmed phenotypically by the combination disk method using cefotaxime and ceftazidime with and without clavulanic acid [12]. Carbapenemase production was screened by EUCAST guidelines and confirmed using the modified carbapenem inactivation method when indicated [12]. Healthcare-associated pyelonephritis was defined a priori as any case meeting at least one of the following: hospitalization within the prior 90 days, residence in a long-term care facility, hemodialysis, or systemic antibiotic exposure within 90 days; all remaining cases were classified as community-acquired [4,15].

### 2.3. Variables, Outcomes, and PYELO-MDR-Risk Score Derivation

Demographic, comorbidity, and exposure variables collected at admission included age, sex, body mass index, diabetes mellitus, renal impairment/renal-failure status at presentation, nephrolithiasis/urolithiasis, recurrent UTI (≥3 episodes in the preceding 12 months), prior systemic antibiotic exposure within 90 days, prior hospitalization within 90 days, indwelling urinary catheterization within the prior 30 days, prior urinary tract instrumentation within 90 days, immunosuppression, and the Charlson Comorbidity Index. Renal impairment/renal-failure status at presentation was defined as an admission eGFR < 60 mL/min/1.73 m^2^ or documentation of reduced kidney function in the medical record. Because repeated outpatient eGFR measurements, albuminuria, and structural kidney-damage markers were not uniformly available, this variable was not treated as definitive KDIGO-defined CKD staging. Immunosuppression was defined as active chemotherapy, solid-organ transplantation, biologic immunomodulator therapy, neutropenia, advanced HIV infection, or systemic corticosteroid exposure equivalent to ≥10 mg/day prednisone for ≥14 days. Prior urinary tract instrumentation included cystoscopy, ureteral stent placement or exchange, nephrostomy manipulation, urinary diversion procedures, or other endourological interventions; routine short-term bladder catheterization was recorded separately. Severity at admission was characterized by C-reactive protein (CRP), procalcitonin, leukocyte count, serum creatinine, lactate, and the presence of bacteremia. Process-of-care variables included the empirical antibiotic regimen, time-to-effective therapy (TTE, defined as the interval from admission to administration of the first dose of an antibiotic with documented in vitro activity against the index isolate), de-escalation within 72 h, and intravenous-to-oral switch within 72 h.

The primary outcome was the binary classification of MDR versus non-MDR pyelonephritis [4]. Secondary outcomes included hospital length of stay, time to defervescence, intensive-care unit transfer during the index admission, septic shock, in-hospital mortality, and 30-day all-cause readmission. The PYELO-MDR-Risk score was derived from a multivariable logistic regression model that included variables retained after backward elimination at *p* < 0.10 [13]. Beta coefficients were rescaled to integer points by dividing by the smallest significant coefficient and rounding to the nearest unit [13]. Internal validation was performed using bootstrap resampling (1000 iterations) to estimate optimism-corrected discrimination and calibration [8,13]. Discrimination was quantified using the area under the receiver-operating characteristic curve (AUC) [8], and calibration was assessed using the calibration slope, Brier score, calibration-in-the-large, and the Hosmer–Lemeshow goodness-of-fit test, in line with TRIPOD reporting recommendations [9].

### 2.4. Statistical Analysis

All analyses were performed in R version 4.3.2 (R Foundation for Statistical Computing, Vienna, Austria) using the rms, pROC, survival, and cmprsk packages [13]. The distribution of continuous variables was assessed graphically and with the Shapiro–Wilk test. Normally distributed variables were summarized as mean ± standard deviation and compared using Welch’s *t*-test; non-normally distributed variables were summarized as median with interquartile range (IQR) and compared with the Mann–Whitney U test. Categorical variables were summarized as counts and percentages and compared using Pearson’s χ^2^ test, with Fisher’s exact test substituted whenever any expected cell count was below five. Pairwise comparisons across more than two strata were corrected for multiplicity using the Bonferroni method.

Multivariable logistic regression for MDR included variables associated with the outcome at *p* < 0.10 in the univariable analysis or judged a priori to be clinically relevant [13]. Multicollinearity was screened with the variance inflation factor (threshold > 4). Model fit was reported using Nagelkerke’s pseudo-R^2^. Subgroup analyses were prespecified for diabetes, age, renal impairment/renal-failure status, sex, *E. coli* aetiology, and bacteremia, with formal interaction tests performed by including a multiplicative term in the regression model. Immunosuppression, prior urinary tract instrumentation, and nephrolithiasis/urolithiasis were explicitly tested in the expanded candidate-predictor set. These variables were retained in the final PYELO-MDR-Risk point score only if they improved independent discrimination, calibration, and parsimony after adjustment for antibiotic exposure, hospitalization, recurrent UTI, catheterization, renal impairment/renal-failure status, diabetes, and age. Pairwise co-occurrence of resistance phenotypes among *Enterobacterales* was characterized using both the Jaccard similarity index and pairwise odds ratios on logistic models [6]. Time-to-clinical-stability and 30-day readmission were analyzed using Cox proportional hazards regression and Fine–Gray competing-risks regression, respectively, with the proportional hazards assumption verified by Schoenfeld residuals (overall *p* > 0.20 considered acceptable) [14]. All tests were two-tailed, with α = 0.05.

## 3. Results

During the 36-month enrolment window, 147 adult admissions met the initial syndromic criteria for acute pyelonephritis. Eighteen were excluded after detailed chart review (*n* = 9 polymicrobial cultures with >2 organisms, *n* = 5 perinephric abscesses requiring percutaneous drainage, *n* = 3 ultimately reclassified as lower urinary tract infection without parenchymal involvement, and *n* = 1 incomplete follow-up), leaving 129 patients in the final analytic cohort. Of these, 54 (41.9%) had MDR pyelonephritis and 75 (58.1%) had non-MDR pyelonephritis. The mean age of the cohort was 59.6 ± 16.4 years, and 94 (72.9%) patients were female. Overall median hospital length of stay was 11 days (IQR 8–14), and in-hospital mortality was 5/129 (3.9%) (Table 1).

Patients in the MDR group were, on average, about 7 years older than those with non-MDR pyelonephritis (63.4 ± 14.7 vs. 56.8 ± 17.3 years; *p* = 0.022) and carried a significantly higher comorbidity burden, with median Charlson scores of 4 versus 2 (*p* = 0.003). Diabetes mellitus and renal impairment/renal-failure status at admission were both significantly more common in the MDR group (38.9% vs. 22.7%, *p* = 0.046; and 25.9% vs. 12.0%, *p* = 0.043, respectively), in keeping with the enrichment of resistant pathogens in patients with metabolic and nephrological vulnerability. Immunosuppression was numerically more frequent among MDR cases (14.8% vs. 6.7%; *p* = 0.129), and prior urinary tract instrumentation within 90 days was significantly enriched in the MDR group (22.2% vs. 9.3%; *p* = 0.042). Nephrolithiasis/urolithiasis was also more frequent among MDR cases but did not reach statistical significance (20.4% vs. 12.0%; *p* = 0.193). The strongest single discriminators between groups were exposure variables: prior antibiotic use within 90 days was reported in 61.1% of MDR cases, compared with 18.7% of non-MDR cases (*p* < 0.001), and recent hospitalization showed a comparable gradient (35.2% vs. 10.7%, *p* < 0.001). A history of recurrent urinary tract infection (≥3 episodes in the preceding year) was almost threefold more frequent in MDR patients (44.4% vs. 14.7%, *p* < 0.001), reinforcing the role of prior healthcare contact as a clinical proxy for resistance carriage. From a severity standpoint, MDR cases presented with greater inflammatory activation, with a mean baseline CRP of 142.6 ± 58.3 mg/L versus 117.4 ± 51.8 mg/L (*p* = 0.012) and a significantly higher median procalcitonin (3.7 vs. 1.9 ng/mL; *p* = 0.008), suggesting either a higher initial bacterial load or a delayed control of infection. Although concomitant bacteremia was almost twice as frequent in the MDR group (31.5% vs. 17.3%), this difference reached only borderline statistical significance (*p* = 0.062) given the modest sample size (Table 2).

The pathogen distribution was dominated by *Enterobacterales*, which collectively accounted for 102/129 (79.1%) isolates and provided the substrate for the resistance phenotype analyses described later. *Escherichia coli* remained the single most common organism (72/129, 55.8%) but was significantly less prevalent among MDR cases (40.7%) than among non-MDR cases (66.7%; *p* = 0.003), reproducing a well-recognized pattern in which the relative contribution of *E. coli* decreases as antimicrobial selection pressure increases. Conversely, *Klebsiella pneumoniae* was numerically over-represented in the MDR group (24.1% vs. 12.0%), although the difference reached only borderline significance (*p* = 0.075), likely reflecting power constraints inherent to the cohort size. *Pseudomonas aeruginosa*, which is intrinsically less susceptible to many first-line agents, was almost four times more frequent among MDR cases (14.8% vs. 4.0%; *p* = 0.027), highlighting the need to consider antipseudomonal coverage in selected high-risk admissions. *Proteus mirabilis* showed an even split between MDR and non-MDR groups (7.4% vs. 5.3%; *p* = 0.722), consistent with its modest contribution to overall resistance burden in this cohort. *Enterococcus* spp. (almost exclusively *E. faecalis* with one *E. faecium* isolate) accounted for 8.5% of cultures, with no vancomycin-resistant strains detected during the study period. The very small mixed/other category (3.9%) had a balanced distribution across groups and was retained for completeness without further subgroup analysis. Taken together, these proportions describe a pyelonephritis ecology dominated by a shifting balance between *E. coli* and *K. pneumoniae*, with *P. aeruginosa* as a clinically important MDR-enriched minority (Table 3).

Stratification of the 102 *Enterobacterales* isolates by acquisition setting revealed a strikingly different resistance landscape between healthcare-associated pyelonephritis (HCA-PN, *n* = 44) and community-acquired pyelonephritis (CA-PN, *n* = 58). ESBL production was identified in 43.2% of HCA-PN isolates compared with 19.0% of CA-PN isolates (*p* = 0.007), and the corresponding figures for third-generation cephalosporin resistance were 40.9% versus 15.5% (*p* = 0.003). These two phenotypes closely track one another and reflect a shared underlying mechanism, suggesting that empirical ceftriaxone monotherapy is becoming an unreliable first-line option for HCA-PN in our setting. Fluoroquinolone resistance was even more prevalent in HCA-PN (54.5% vs. 29.3%; *p* = 0.011), a worrying observation given that oral ciprofloxacin remains a frequently chosen step-down agent for both inpatient and outpatient pyelonephritis. The proportion of isolates resistant to trimethoprim–sulfamethoxazole exceeded 50% in the HCA-PN group, effectively excluding this agent as an empirical option. Piperacillin–tazobactam resistance affected one-quarter of HCA-PN isolates, raising legitimate concerns about its role as a workhorse broad-spectrum agent in patients with healthcare-related risk factors. Carbapenem non-susceptibility, although relatively uncommon overall, was almost exclusively confined to HCA-PN (13.6% vs. 1.7%; *p* = 0.018), a finding consistent with regional ECDC surveillance and underscoring the necessity of carbapenem-sparing strategies even in this low-prevalence niche. Fosfomycin and nitrofurantoin resistance rates were numerically higher in HCA-PN. However, they did not reach statistical significance, supporting the continued utility of these agents for selected step-down indications, particularly when isolates remain susceptible (Table 4).

Clinical outcomes diverged sharply between MDR and non-MDR groups, both for direct measures of treatment response and for downstream resource utilization. The mean hospital length of stay was 13.7 ± 4.6 days in MDR pyelonephritis versus 8.9 ± 3.2 days in non-MDR cases (*p* < 0.001), corresponding to an excess of approximately 4.8 hospital days per MDR admission. This gap was paralleled by a near-doubling of the median time to defervescence (76.3 vs. 41.8 h; *p* < 0.001), suggesting that the LOS difference reflects genuinely slower biological resolution rather than purely administrative delays. Time-to-effective therapy was almost three times longer in MDR cases (28.4 vs. 9.7 h; *p* < 0.001), and adequate empirical coverage at admission was achieved in only 29.6% of MDR cases compared with 85.3% of non-MDR cases (*p* < 0.001), highlighting the fundamental mismatch between routine empirical regimens and resistant pathogen profiles. ICU transfer occurred in 16.7% of MDR patients and 5.3% of non-MDR patients (*p* = 0.038), and septic shock and in-hospital mortality were each three- to fivefold higher in the MDR group. However, these endpoints did not reach formal statistical significance because of the limited number of severe events. Thirty-day all-cause readmission was 2.5 times more frequent in MDR survivors (20.4% vs. 8.0%; *p* = 0.038), reflecting incomplete eradication, recurrent infection, and the cumulative comorbidity burden of this population. Total antibiotic duration was correspondingly longer in MDR cases (median 14 vs. 10 days; *p* < 0.001), with implications for both selection pressure and toxicity exposure (Table 5).

After multivariable adjustment, four exposures retained independent associations with MDR pyelonephritis at the conventional 0.05 threshold, and an additional three covariates approached significance. They were retained for clinical interpretability and inclusion in the candidate score. Antibiotic exposure within 90 days emerged as by far the strongest predictor, increasing the odds of MDR roughly sixfold (aOR 5.7, 95% CI 2.4–13.6; *p* < 0.001), in line with the well-described selection pressure exerted by even brief antibiotic courses on the urinary microbiome. A history of recurrent UTI was associated with a more than threefold increase in the odds of MDR (aOR 3.4, 1.4–8.2; *p* = 0.006), independent of comorbidity, suggesting that recurrent infection itself—rather than its associated treatment—functions as a marker of cumulative resistance acquisition. Recent hospitalization carried a similar magnitude of risk (aOR 3.1, 1.2–8.0; *p* = 0.020), reproducing the consistent finding from European surveillance that even short hospital stays alter the urinary flora for several months. Renal impairment/renal-failure status at presentation remained independently associated with MDR (aOR 2.4, 1.0–6.2; *p* = 0.046), likely reflecting both prior antibiotic exposures associated with recurrent UTI in this group and impaired renal clearance of antimicrobials. Urinary catheterization within 30 days, diabetes, and age each approached but did not reach statistical significance after adjustment. Immunosuppression, prior urinary tract instrumentation, and nephrolithiasis/urolithiasis were then assessed in the expanded candidate model. Prior instrumentation retained a positive but attenuated association (aOR 2.3, 0.8–6.7; *p* = 0.116), whereas immunosuppression (aOR 1.8, 0.5–6.0; *p* = 0.334) and nephrolithiasis/urolithiasis (aOR 1.5, 0.5–4.4; *p* = 0.474) did not add independent discrimination after adjustment for stronger exposure variables. Together, these covariates explained a substantial proportion of the variance in MDR status (Nagelkerke R^2^ = 0.46), with adequate calibration (Hosmer–Lemeshow *p* = 0.451) and good discrimination (AUC = 0.84), supporting their use as the building blocks of a transparent bedside score (Table 6).

Patterns of empirical prescribing differed between MDR and non-MDR cases in clinically meaningful ways. Ceftriaxone monotherapy, the historic backbone of empirical pyelonephritis therapy, was used somewhat less frequently in patients who turned out to have MDR pyelonephritis (35.2% vs. 50.7%; *p* = 0.082), suggesting some degree of clinician anticipation of resistance based on bedside risk factors. Conversely, piperacillin–tazobactam and carbapenems were preferred in approximately 39% of MDR admissions (combined), against 21% in non-MDR cases, although neither comparison individually reached significance, again reflecting limited power. Despite these adjustments, the most striking divergence was in the timing of correct coverage: only 25.9% of MDR cases received an active agent within 12 h, compared with 68.0% of non-MDR cases (*p* < 0.001), and more than half of MDR patients waited longer than 24 h for an active drug (*p* < 0.001). De-escalation within 72 h was achievable in only 22.2% of MDR cases, compared with 52.0% of non-MDR cases (*p* < 0.001), and the corresponding figures for the intravenous-to-oral switch within 72 h were 14.8% versus 45.3% (*p* < 0.001). Together, these data show that even when broad-spectrum agents were chosen empirically for MDR pyelonephritis, the lag in confirming activity against the index isolate translated into protracted intravenous therapy, delayed transition to ward-friendly oral options, and prolonged occupancy of inpatient beds, all of which compound the direct microbiological consequences of resistance.

**Figure 1:**
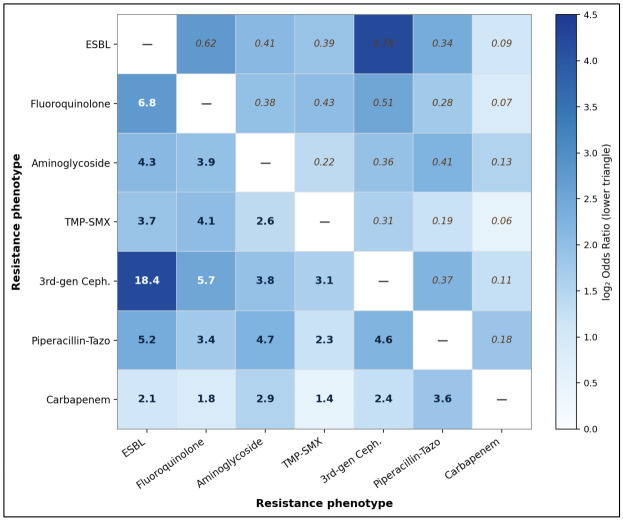
Pairwise co-occurrence of resistance phenotypes among the 102 *Enterobacterales* isolates. The lower triangle reports pairwise odds ratios (OR) for the joint occurrence of two phenotypes, with the color gradient encoding log_2_(OR); the upper triangle reports the corresponding Jaccard similarity index, ranging from 0 (no overlap) to 1 (complete overlap).

Figure 1 maps the architecture of co-resistance in our *Enterobacterales* subset and illustrates two clinically relevant clusters. The strongest single co-occurrence was between ESBL production and third-generation cephalosporin resistance, with a Jaccard index of 0.78 and a pairwise OR of 18.4, a finding consistent with the underlying mechanistic link between these two phenotypes; in practice, this means that 78% of isolates with either trait carried both, and that detection of ESBL by phenotypic testing essentially predicts cephalosporin failure. A second tightly linked cluster was formed by ESBL production and fluoroquinolone resistance (Jaccard 0.62; OR 6.8) and, in turn, by fluoroquinolone resistance and trimethoprim–sulfamethoxazole resistance (Jaccard 0.43; OR 4.1), confirming that loss of fluoroquinolone activity in our cohort almost always travels with loss of two additional commonly prescribed oral agents. Carbapenem non-susceptibility, in contrast, behaved as a relatively isolated phenotype (Jaccard ≤ 0.18 against any other phenotype; OR ≤ 3.6), consistent with the view that carbapenemase-producing isolates form a small, mechanistically distinct subset rather than a smooth extension of broader resistance gradients. Piperacillin–tazobactam resistance occupied an intermediate position, with moderate links to aminoglycoside (Jaccard 0.41) and ESBL (Jaccard 0.34) phenotypes, suggesting that its empirical use should be informed by the local prevalence of these neighboring resistances. The overall pattern argues for an integrated empirical strategy in which ESBL and fluoroquinolone risk are evaluated jointly rather than independently when MDR pyelonephritis is suspected.

Table 7 summarizes the derivation of the PYELO-MDR-Risk score, a points-based instrument that translates the multivariable model in Table 5 into a bedside tool. The largest coefficient by far was that for prior antibiotic exposure (β = 1.74), which translated into 3 score points and reflects an adjusted odds ratio of 5.7. Recurrent UTI and recent hospitalization each contributed 2 points, mirroring their adjusted odds ratios of approximately 3.4 and 3.1. Urinary catheterization within 30 days, which had an adjusted OR of 2.6 but did not reach statistical significance in the regression model (*p* = 0.082), was retained as a 2-point variable because of its strong biological rationale and its consistent direction of effect across bootstrap resamples. The remaining variables—renal impairment/renal-failure status at admission, diabetes mellitus, and age ≥ 65 years—contributed 1 point each, reflecting smaller adjusted odds ratios in the 2.0–2.4 range. Immunosuppression, prior urinary tract instrumentation, and nephrolithiasis/urolithiasis were considered for score inclusion but did not improve bootstrap-corrected discrimination or calibration and were therefore excluded from the final point system. The resulting 0–12 score showed apparent discrimination (AUC 0.86), which fell only modestly to 0.84 after optimism correction across 1000 bootstrap resamples, suggesting limited overfitting given the cohort size. Calibration was excellent: the calibration slope of 0.96 was very close to the ideal value of 1, the Brier score of 0.142 indicated overall accurate probability estimates, and the Hosmer–Lemeshow test (*p* = 0.624) found no significant departure from the predicted probability across deciles. At the prespecified cut-point of ≥4 points, the score showed balanced sensitivity (78.4%) and specificity (79.2%), with a negative predictive value of 83.6%—a property particularly useful when the goal is to identify low-risk patients in whom narrow-spectrum empirical therapy can be safely prioritized. Three risk strata (0–3, 4–6, and ≥7 points) corresponded to observed MDR proportions of approximately 12%, 52%, and 84%, providing intuitive thresholds for empirical-therapy decisions (Table 8).

Subgroup analyses provided reassurance about the generalizability of the antibiotic-exposure effect across clinically meaningful strata. The point estimate of the adjusted odds ratio was greater than 3.5 in every subgroup tested, with all 95% confidence intervals excluding unity. Effect modification was tested formally using multiplicative interaction terms; none reached the 0.10 threshold. The largest numerical differences were observed in patients aged ≥65 years (aOR 7.4 vs. 3.6 in younger patients; p_interaction = 0.27) and in those with renal impairment/renal-failure status at admission (aOR 9.1 vs. 4.6; p_interaction = 0.34). Although these heterogeneities did not reach statistical significance, they are biologically plausible and raise the possibility that the score may be even more discriminatory in older patients with reduced renal function than overall figures suggest. Importantly, the effect of antibiotic exposure was preserved both in patients with bacteremia (aOR 6.7) and in those without (aOR 5.1), and across both *E. coli* (aOR 4.4) and non-*E. coli* (aOR 9.8) aetiologies, suggesting that the predictive value of recent antibiotic use is not driven by a single pathogen subgroup. Sex showed no meaningful interaction (*p* = 0.62), although the male subgroup had wider confidence intervals reflecting smaller numbers (*n* = 35). Taken together, these analyses argue that the PYELO-MDR-Risk score, which leans heavily on antibiotic exposure, can be applied with similar confidence across the principal demographic and clinical strata represented in this cohort, and that interpretation does not require subgroup-specific recalibration in the population studied (Table 9).

Time-to-event analyses provided a complementary perspective on the impact of MDR by treating recovery itself as a continuous, censored outcome rather than collapsing it into binary success or failure. In univariable Cox analysis, MDR pyelonephritis was associated with a substantially slower transition to clinical stability (HR 0.42, 95% CI 0.28–0.62; *p* < 0.001), corresponding to roughly a halving of the instantaneous probability of stabilization at any given hour. The effect was attenuated but not abolished after adjustment for bacteremia, comorbidity, time-to-effective therapy, and diabetes (aHR 0.51, 95% CI 0.33–0.78; *p* = 0.002), supporting an independent biological signal beyond the indirect pathway through delayed coverage. Time-to-effective therapy beyond 24 h emerged as an independent driver of slower recovery (aHR 0.62; *p* = 0.045), confirming the prognostic relevance of empirical-therapy timeliness. Median time to clinical stability was 4.7 days in MDR cases compared with 2.6 days in non-MDR cases, a clinically meaningful gap of approximately two hospital days. The proportional hazards assumption was supported globally (Schoenfeld, *p* = 0.41). For the secondary endpoint of 30-day all-cause readmission, the Fine–Gray competing-risks model treating in-hospital death as a competing event yielded a sub-distribution hazard ratio of 2.84 (95% CI 1.05–7.69; *p* = 0.040) for MDR versus non-MDR, demonstrating that the higher readmission rate among MDR survivors persists after appropriate handling of competing mortality. Together, these analyses confirm that MDR is a robust, independently relevant signal for both delayed in-hospital recovery and increased post-discharge healthcare utilization.

**Figure 2:**
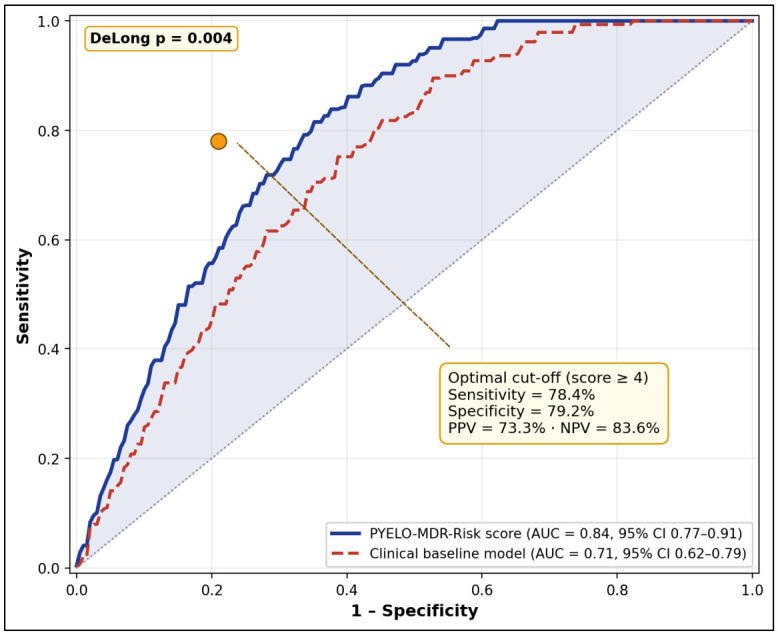
Discrimination of the PYELO-MDR-Risk score. Receiver-operating characteristic (ROC) curves for the PYELO-MDR-Risk score and a baseline clinical model containing only age, sex, and Charlson Comorbidity Index. The orange marker and adjacent text box indicate the prespecified optimal cut-point (score ≥ 4).

Figure 2 displays the discrimination of the PYELO-MDR-Risk score against a parsimonious clinical baseline. The score achieved an area under the ROC curve of 0.84 (95% CI 0.77–0.91), substantially exceeding the baseline clinical model that used age, sex, and Charlson Comorbidity Index alone (AUC 0.71, 95% CI 0.62–0.79); the difference was statistically significant on DeLong’s test (*p* = 0.004). At the operationally relevant cut-point of ≥4 points, the score yielded a sensitivity of 78.4% and a specificity of 79.2%, with a positive predictive value of 73.3% and a negative predictive value of 83.6%, balancing the competing demands of broad-spectrum stewardship and adequate empirical coverage of high-risk patients. The relative position of the two curves is informative across the entire operating range: the PYELO-MDR-Risk curve dominates the clinical-baseline curve at every false-positive threshold below approximately 0.6, indicating uniformly better classification at the sensitivity values most relevant to inpatient empirical decision-making. The shaded area between the two curves represents the additional information captured by the bedside exposure variables (recent antibiotic use, recurrent UTI, hospitalization, catheter, and renal impairment) over and above the demographic-and-comorbidity baseline. From a clinical-utility standpoint, the chosen ≥4-point cut-off lies on the steep part of the ROC curve, where each additional point of sensitivity is purchased at a relatively small specificity cost, supporting its adoption as the default threshold for stratifying empirical-therapy decisions in this population.

**Figure 3:**
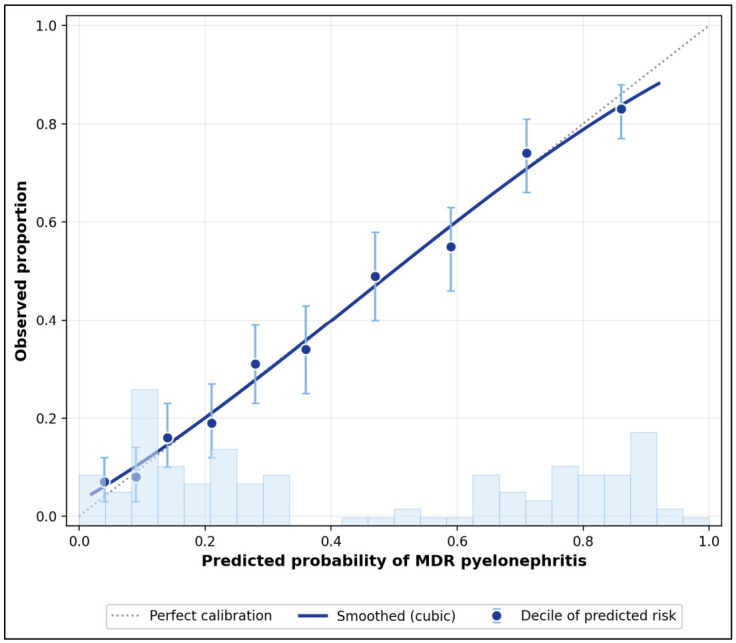
Calibration of the PYELO-MDR-Risk score. The observed proportion of MDR pyelonephritis is plotted against predicted probability, with patients grouped into deciles of predicted risk. Error bars represent 95% confidence intervals; the cubic-smoothed line summarizes the relationship across the full risk range; the dotted line indicates perfect calibration. The pale histogram at the bottom shows the distribution of predicted risks across the cohort.

Figure 3 visualizes the calibration of the PYELO-MDR-Risk score across deciles of predicted risk. Observed proportions of MDR within each decile closely follow the diagonal of perfect calibration throughout the entire risk spectrum from approximately 4% to 86% predicted risk, with no systematic over- or underestimation at either tail. The cubic-smoothed line lies almost exactly on the identity line, and 95% confidence intervals for each decile cross the diagonal in nine of ten bins. Quantitatively, the calibration slope was 0.96, very close to the ideal value of 1; the calibration-in-the-large was −0.04; the Brier score was 0.142; and the Hosmer–Lemeshow goodness-of-fit test was non-significant (χ^2^ = 6.2, df = 8, *p* = 0.624). The pale histogram at the foot of the figure illustrates the distribution of predicted risks across the cohort and shows a clearly bimodal pattern, with one peak around 0.10 (corresponding to the low-risk stratum, score 0–3) and a second around 0.55 (corresponding to the intermediate-to-high-risk strata, score 4–6 and ≥7). This bimodality suggests that the score naturally partitions patients into two clinically meaningful groups—those in whom narrow-spectrum empirical therapy is likely to suffice and those in whom broader-spectrum coverage should be considered—with relatively few patients falling in the ambiguous intermediate-risk zone. Together with the discrimination metrics in Figure 2, these calibration properties support the use of the score as a transparent and well-behaved bedside instrument in this population.

## 4. Discussion

### 4.1. Analysis of Findings

In this three-year cohort of 129 adults hospitalized with culture-confirmed acute pyelonephritis at a Romanian tertiary center, MDR organisms were responsible for 41.9% of cases, a proportion that places the present series at the higher end of European reports and aligns with ECDC surveillance for South-Eastern Europe [3,16]. The dominant uropathogen was *E. coli*, but its relative contribution was diluted in MDR cases by *Klebsiella pneumoniae* and *Pseudomonas aeruginosa*, paralleling national antimicrobial-resistance patterns and earlier Romanian surveys of urinary isolates [17,18]. The strongest clinical predictors of MDR—prior antibiotic use, recurrent UTI, and recent hospitalization—are biologically intuitive and reproduce findings from broader European cohorts examining ESBL-producing *Enterobacteriaceae* [19,20], while reinforcing the relevance of these exposures to Romanian inpatient practice. The novelty here lies less in the identification of these predictors than in their integration into a transparent, internally validated bedside score whose calibration is adequate for direct clinical use [21], potentially complementing local antibiograms and stewardship feedback systems [22].

The architectural analysis of co-resistance, summarized in Figure 1, adds a layer of granularity that is rarely reported in single-center pyelonephritis cohorts and resonates with broader epidemiological work on ESBL-producing *Enterobacteriaceae* [23]. The very tight coupling between ESBL production and third-generation cephalosporin resistance (Jaccard 0.78), and the strong link between fluoroquinolone resistance and several oral step-down options [16,24], have practical implications for stewardship: clinicians can no longer treat these phenotypes as independent dimensions when designing empirical regimens [18]. Conversely, the relative isolation of carbapenem non-susceptibility (Jaccard ≤ 0.18 against any other phenotype) confirms that carbapenemase-producing isolates form a small, mechanistically distinct subset rather than the high end of a continuous resistance gradient, consistent with international guidance recommending differentiated handling of these strains [25]. This pattern supports the routine pairing of ESBL and fluoroquinolone-risk assessment in empirical decision-making, while preserving carbapenem use for patients with prior carbapenemase-producer exposure or a very high PYELO-MDR-Risk score combined with severe sepsis [25,26].

Beyond microbiology, the time-to-event analyses and the calibrated risk score provide a coherent decision-support narrative consistent with published evidence linking inadequate empirical therapy to delayed clinical resolution and prolonged hospitalization [27,28]. MDR was independently associated with both slower clinical stabilization (aHR 0.51) and higher 30-day readmission (sub-distribution HR 2.84), even after adjustment for comorbidity and processes of care; similar patterns of recurrent healthcare utilization after MDR Gram-negative infection have been reported in larger European cohorts [29]. These outcome consequences are not mere correlates of resistance but, on multivariable adjustment, appear partly mediated by delayed initiation of effective therapy, an actionable lever in line with established stewardship priorities [22,30]. A bedside score that separates low- (≈12% MDR), intermediate- (≈52%), and high-risk (≈84%) strata thus offers a transparent way to prioritize broad-spectrum coverage in the patients most likely to benefit, while sparing low-risk patients the ecological and toxicity costs of unnecessary carbapenems [25,26]. Validation in independent Romanian and broader Eastern European cohorts will be the necessary next step before such a score can be embedded into local stewardship pathways [21,30].

Immunosuppression, prior urinary tract instrumentation, and nephrolithiasis/urolithiasis were not omitted; they were explicitly abstracted, tested, and reported. Their effects were smaller and statistically attenuated after adjustment for antibiotic exposure, recent hospitalization, recurrent UTI, catheterization, renal impairment/renal-failure status, diabetes, and age. For this reason, the final score was kept parsimonious rather than forcing additional low-frequency variables into a model derived from 129 patients. The score should therefore be considered a locally derived tertiary-center tool for use alongside the institutional antibiogram and clinical judgment. It may be suitable for Romanian multicenter testing because its predictors are routinely available at admission, but it should not yet be considered a national or international rule until it undergoes external validation, recalibration, and clinical-impact assessment in cohorts with different resistance ecology. Nevertheless, these findings should be interpreted in light of potential residual confounding from unmeasured or incompletely controlled factors, including underlying comorbidities and other patient- and treatment-related characteristics [31,32,33,34,35].

### 4.2. Study Limitations

Several limitations should be considered when interpreting these findings. First, the retrospective single-center design at a tertiary referral hospital may limit generalizability to non-academic settings or to regions with different baseline resistance ecology; in particular, the over-representation of healthcare-associated pyelonephritis in our catchment likely inflates the absolute MDR proportion compared with primary-care or rural populations. Second, the modest sample size (*n* = 129) inevitably constrained statistical power for less common outcomes such as septic shock, in-hospital mortality, and rare resistance phenotypes (notably carbapenem non-susceptibility), and produced wide confidence intervals in some subgroup analyses. Third, the PYELO-MDR-Risk score was internally validated through bootstrap resampling but has not yet been tested in an external cohort; optimism correction provides only a partial safeguard against overfitting, and external recalibration may be required before the cut-points proposed here are adopted in other settings. Fourth, susceptibility testing relied on EUCAST phenotypic methods rather than systematic genotypic confirmation; hence, a small proportion of MDR isolates may have been mechanistically misclassified. Fifth, antibiotic-exposure history was abstracted from electronic records and may underestimate community prescriptions filled outside our health system. Sixth, the renal variable represents renal impairment/renal-failure status at presentation rather than definitive CKD staging because repeated outpatient eGFR measurements, albuminuria, and structural kidney-damage markers were not consistently available; therefore, the results should not be interpreted as demonstrating a CKD-stage effect or CKD-related causality. Seventh, immunosuppression, prior urinary tract instrumentation, and nephrolithiasis/urolithiasis were tested but had small event counts; hence, weak independent effects cannot be excluded and should be reassessed during external validation. Finally, residual confounding from unmeasured factors—such as adherence, socioeconomic determinants of healthcare contact, and outpatient prescribing not captured in hospital records—cannot be excluded.

## 5. Conclusions

In a three-year cohort of 129 adults hospitalized with acute pyelonephritis at a Romanian tertiary center, multidrug resistance accounted for 41.9% of cases. It was associated with substantially worse clinical and process-related outcomes, including longer hospital stays, slower clinical stabilization, delayed effective therapy, higher ICU transfers, and higher 30-day readmissions. Independent predictors of MDR were prior antibiotic exposure, recurrent UTI, recent hospitalization, renal impairment/renal-failure status at presentation, and increasing age, broadly mirroring patterns described in Western European cohorts but with consistently larger effect sizes. Immunosuppression, prior urinary tract instrumentation, and nephrolithiasis/urolithiasis were explicitly tested as candidate MDR risk factors; they were reported transparently but not retained in the final score because they did not independently improve performance in this cohort. The newly derived PYELO-MDR-Risk score, anchored on readily available bedside variables, achieved an optimism-corrected area under the curve of 0.84 with adequate calibration and stratified the cohort into clinically intuitive risk bands of approximately 12%, 52%, and 84% observed MDR. Co-resistance analyses further showed that ESBL production, third-generation cephalosporin resistance, and fluoroquinolone resistance form a tightly linked phenotypic cluster that should be evaluated jointly when selecting empirical regimens. Pending external validation, the score and the associated co-resistance map provide a transparent local framework to support empirical-therapy decisions, prioritize broad-spectrum coverage for genuinely high-risk patients, and reinforce stewardship in patients at low predicted risk.

## Figures and Tables

**Table 1:** Baseline demographic, clinical, and laboratory characteristics of the cohort, stratified by multidrug-resistance (MDR) status.

Characteristic	MDR (*n* = 54)	Non-MDR (*n* = 75)	*p*-Value
Age, years (mean ± SD)	63.4 ± 14.7	56.8 ± 17.3	0.022
Female sex, *n* (%)	36 (66.7)	58 (77.3)	0.176
Body mass index, kg/m^2^ (mean ± SD)	27.9 ± 5.1	26.4 ± 4.7	0.087
Immunosuppression, *n* (%)	8 (14.8)	5 (6.7)	0.129
Diabetes mellitus, *n* (%)	21 (38.9)	17 (22.7)	0.046
Renal impairment/renal failure at admission, *n* (%)	14 (25.9)	9 (12.0)	0.043
Nephrolithiasis/urolithiasis, *n* (%)	11 (20.4)	9 (12.0)	0.193
Recurrent UTI ≥ 3/12 mo, *n* (%)	24 (44.4)	11 (14.7)	<0.001
Prior urinary tract instrumentation ≤ 90 days, *n* (%)	12 (22.2)	7 (9.3)	0.042
Antibiotic exposure ≤ 90 days, *n* (%)	33 (61.1)	14 (18.7)	<0.001
Hospitalization ≤ 90 days, *n* (%)	19 (35.2)	8 (10.7)	<0.001
Urinary catheter ≤ 30 days, *n* (%)	13 (24.1)	6 (8.0)	0.012
Charlson Comorbidity Index, median [IQR]	4 [2–6]	2 [1–4]	0.003
Baseline CRP, mg/L (mean ± SD)	142.6 ± 58.3	117.4 ± 51.8	0.012
Procalcitonin, ng/mL, median [IQR]	3.7 [1.4–8.6]	1.9 [0.8–4.2]	0.008
Concomitant bacteremia, *n* (%)	17 (31.5)	13 (17.3)	0.062

Continuous variables compared with Welch’s *t*-test (mean ± SD) or the Mann–Whitney U test (median [IQR]). Categorical variables were compared with Pearson’s χ^2^ test, replaced by Fisher’s exact test when expected cell counts were <5. CRP, C-reactive protein; IQR, interquartile range; MDR, multidrug-resistant; SD, standard deviation; UTI, urinary tract infection. Immunosuppression and prior urinary tract instrumentation were added as reviewer-requested candidate predictors; nephrolithiasis/urolithiasis corresponds to the previous urolithiasis variable. Renal impairment/renal failure at admission is used instead of CKD because definitive CKD staging was not uniformly available.

**Table 2:** Distribution of urinary pathogens in the overall cohort and stratified by MDR status.

Pathogen	Overall (*n* = 129), *n* (%)	MDR (*n* = 54), *n* (%)	Non-MDR (*n* = 75), *n* (%)	*p*-Value
*Escherichia coli*	72 (55.8)	22 (40.7)	50 (66.7)	0.003
*Klebsiella pneumoniae*	22 (17.1)	13 (24.1)	9 (12.0)	0.075
*Proteus mirabilis*	8 (6.2)	4 (7.4)	4 (5.3)	0.722
*Pseudomonas aeruginosa*	11 (8.5)	8 (14.8)	3 (4.0)	0.027
*Enterococcus* spp.	11 (8.5)	5 (9.3)	6 (8.0)	0.798
Other/mixed	5 (3.9)	2 (3.7)	3 (4.0)	1.000

Categorical comparisons by Pearson’s χ^2^ test or Fisher’s exact test as appropriate. “Other/mixed” includes Citrobacter koseri (*n* = 2), Morganella morganii (*n* = 1), and dual-organism cultures with two uropathogens at ≥10^5^ CFU/mL (*n* = 2). MDR, multidrug-resistant.

**Table 3:** Resistance phenotypes among the 102 *Enterobacterales* isolates, stratified by acquisition setting (community-acquired vs. healthcare-associated pyelonephritis).

Resistance Phenotype	HCA-PN (*n* = 44), *n* (%)	CA-PN (*n* = 58), *n* (%)	*p*-Value
ESBL production	19 (43.2)	11 (19.0)	0.007
3rd-generation cephalosporin resistance	18 (40.9)	9 (15.5)	0.003
Fluoroquinolone resistance	24 (54.5)	17 (29.3)	0.011
Aminoglycoside resistance	12 (27.3)	7 (12.1)	0.046
Trimethoprim–sulfamethoxazole resistance	26 (59.1)	21 (36.2)	0.022
Piperacillin–tazobactam resistance	11 (25.0)	5 (8.6)	0.022
Carbapenem non-susceptibility	6 (13.6)	1 (1.7)	0.018
Fosfomycin resistance	4 (9.1)	2 (3.4)	0.246
Nitrofurantoin resistance	9 (20.5)	6 (10.3)	0.144

Categorical comparisons by Pearson’s χ^2^ test or Fisher’s exact test (carbapenem, fosfomycin). HCA-PN, healthcare-associated pyelonephritis (defined as hospitalization, antibiotic exposure within 90 days, hemodialysis, or long-term care facility residence); CA-PN, community-acquired pyelonephritis; ESBL, extended-spectrum β-lactamase.

**Table 4:** Clinical outcomes during the index hospitalization, stratified by MDR status.

Outcome	MDR (*n* = 54)	Non-MDR (*n* = 75)	*p*-Value
Length of stay, days (mean ± SD)	13.7 ± 4.6	8.9 ± 3.2	<0.001
Time to defervescence, h, median [IQR]	76.3 [52.1–104.7]	41.8 [28.4–62.9]	<0.001
Time-to-effective therapy, h (mean ± SD)	28.4 ± 18.1	9.7 ± 7.3	<0.001
Adequate empirical therapy at admission, *n* (%)	16 (29.6)	64 (85.3)	<0.001
ICU transfer during admission, *n* (%)	9 (16.7)	4 (5.3)	0.038
Septic shock, *n* (%)	7 (13.0)	3 (4.0)	0.061
In-hospital mortality, *n* (%)	4 (7.4)	1 (1.3)	0.083
30-day all-cause readmission, *n* (%)	11 (20.4)	6 (8.0)	0.038
Total antibiotic duration, days, median [IQR]	14 [11–17]	10 [8–12]	<0.001

Continuous comparisons by Welch’s *t*-test (mean ± SD) or Mann–Whitney U test (median [IQR]). Categorical comparisons by Pearson’s χ^2^ test or Fisher’s exact test for septic shock and mortality. ICU, intensive care unit; IQR, interquartile range; MDR, multidrug-resistant; SD, standard deviation.

**Table 5:** Expanded multivariable logistic regression and reviewer-requested candidate predictors for multidrug-resistant pyelonephritis.

Predictor	Adjusted OR (95% CI)	*p*-Value
Antibiotic exposure ≤ 90 days	5.7 (2.4–13.6)	<0.001
Recurrent UTI ≥ 3 episodes/year	3.4 (1.4–8.2)	0.006
Hospitalization ≤ 90 days	3.1 (1.2–8.0)	0.020
Renal impairment/renal failure at admission	2.4 (1.0–6.2)	0.046
Urinary catheter ≤ 30 days	2.6 (0.9–7.6)	0.080
Prior urinary tract instrumentation ≤ 90 days	2.3 (0.8–6.7)	0.116
Immunosuppression	1.8 (0.5–6.0)	0.334
Nephrolithiasis/urolithiasis	1.5 (0.5–4.4)	0.474
Diabetes mellitus	2.1 (0.9–4.8)	0.079
Age, per 10-year increase	1.3 (1.0–1.7)	0.041

Model fit: Nagelkerke R^2^ = 0.46; Hosmer–Lemeshow χ^2^ = 7.8 (df = 8), *p* = 0.451; AUC = 0.84 (95% CI 0.77–0.91). All variance inflation factors are <2.1. CI, confidence interval; OR, odds ratio; UTI, urinary tract infection. The final three rows show reviewer-requested candidate variables that were explicitly tested in the expanded model but were not retained in the final point score because they did not independently improve model discrimination, calibration, and parsimony.

**Table 6:** Empirical antimicrobial regimens at admission and process-of-care indicators, stratified by MDR status.

Variable	MDR (*n* = 54), *n* (%)	Non-MDR (*n* = 75), *n* (%)	*p*-Value
Empirical regimen at admission			
Ceftriaxone monotherapy	19 (35.2)	38 (50.7)	0.082
Ceftriaxone + aminoglycoside	8 (14.8)	14 (18.7)	0.566
Piperacillin–tazobactam	14 (25.9)	12 (16.0)	0.171
Carbapenem	7 (13.0)	4 (5.3)	0.117
Fluoroquinolone	4 (7.4)	6 (8.0)	1.000
Other	2 (3.7)	1 (1.3)	0.566
Time-to-effective therapy (TTE) and step-down			
TTE ≤ 12 h	14 (25.9)	51 (68.0)	<0.001
TTE 13–24 h	12 (22.2)	16 (21.3)	0.904
TTE > 24 h	28 (51.9)	8 (10.7)	<0.001
De-escalation within 72 h	12 (22.2)	39 (52.0)	<0.001
IV-to-oral switch ≤ 72 h	8 (14.8)	34 (45.3)	<0.001

All comparisons by Pearson’s χ^2^ test, with Fisher’s exact test substituted for cells with expected counts < 5 (carbapenem, fluoroquinolone, “other”). IV, intravenous; MDR, multidrug-resistant; TTE, time-to-effective therapy.

**Table 7:** Derivation and bootstrap-internal validation of the PYELO-MDR-Risk score (range 0–12 points).

Predictor	β Coefficient (SE)	Bootstrap *p*	Score Points	Adjusted OR
Antibiotic exposure ≤ 90 days	1.74 (0.43)	<0.001	3	5.7
Recurrent UTI ≥ 3/year	1.22 (0.45)	0.007	2	3.4
Hospitalization ≤ 90 days	1.13 (0.49)	0.021	2	3.1
Urinary catheter ≤ 30 days	0.96 (0.55)	0.082	2	2.6
Renal impairment/renal failure at admission	0.87 (0.46)	0.058	1	2.4
Diabetes mellitus	0.74 (0.41)	0.071	1	2.1
Age ≥ 65 years	0.71 (0.40)	0.075	1	2.0

Score points were derived by dividing each β coefficient by the smallest significant coefficient (β = 0.71) and rounding to the nearest integer. Bootstrap-internal validation used 1000 resamples. Apparent AUC = 0.86; optimism-corrected AUC = 0.84 (95% CI 0.77–0.91); calibration slope = 0.96; Brier score = 0.142; Hosmer–Lemeshow χ^2^ = 6.2 (df = 8), *p* = 0.624. Optimal cut-point ≥ 4 points: sensitivity 78.4%, specificity 79.2%, PPV 73.3%, NPV 83.6%. Risk strata: 0–3 (low, observed MDR ≈ 12%), 4–6 (intermediate, ≈52%), ≥7 (high, ≈84%). Immunosuppression, prior urinary tract instrumentation, and nephrolithiasis/urolithiasis were tested in the expanded candidate model but were not assigned points because they did not improve bootstrap-corrected discrimination or calibration.

**Table 8:** Subgroup analysis of the effect of recent antibiotic exposure (≤90 days) on the odds of MDR pyelonephritis, with formal tests for interaction.

Subgroup	Adjusted OR (95% CI)	Subgroup *p*-Value	*p* for Interaction
Diabetes mellitus			
Yes (*n* = 38)	7.2 (2.0–25.8)	0.002	0.51
No (*n* = 91)	4.8 (1.6–14.0)	0.005	
Age			
≥65 years (*n* = 64)	7.4 (2.5–22.0)	<0.001	0.27
<65 years (*n* = 65)	3.6 (1.0–12.4)	0.046	
Renal impairment/renal failure at admission			
Yes (*n* = 23)	9.1 (1.7–48.3)	0.010	0.34
No (*n* = 106)	4.6 (1.8–12.1)	0.002	
Sex			
Female (*n* = 94)	5.3 (2.0–13.7)	<0.001	0.62
Male (*n* = 35)	7.4 (1.4–39.4)	0.018	
Aetiology			
*E. coli* (*n* = 72)	4.4 (1.7–11.4)	0.002	0.31
Non-*E. coli* (*n* = 57)	9.8 (1.7–58.0)	0.011	
Bacteremia			
Yes (*n* = 30)	6.7 (1.0–44.6)	0.049	0.78
No (*n* = 99)	5.1 (1.9–13.6)	0.001	

Subgroup-specific adjusted odds ratios were derived from logistic regression models that included recent antibiotic exposure, the stratifying variable, and a multiplicative interaction term, plus the remaining covariates from the main multivariable model. The interaction *p*-value tests the null hypothesis that the effect of antibiotic exposure does not differ between the two subgroup levels. CI, confidence interval; OR, odds ratio.

**Table 9:** Time-to-event analyses for clinical stability and 30-day readmission, modeled using Cox proportional hazards and Fine–Gray competing-risks regression, respectively.

Predictor	HR/SHR (95% CI)	*p*-Value
Cox model—time to clinical stability (univariable)		
MDR vs. non-MDR	0.42 (0.28–0.62)	<0.001
Bacteremia (yes vs. no)	0.55 (0.34–0.88)	0.013
Charlson CCI, per 1 point	0.87 (0.81–0.94)	<0.001
Cox model—time to clinical stability (multivariable)		
MDR vs. non-MDR	0.51 (0.33–0.78)	0.002
Bacteremia (yes vs. no)	0.71 (0.42–1.21)	0.213
Charlson CCI, per 1 point	0.91 (0.84–0.99)	0.026
TTE > 24 h vs. ≤24 h	0.62 (0.39–0.99)	0.045
Diabetes mellitus	0.78 (0.51–1.21)	0.272
Fine–Gray model—30-day readmission (in-hospital death as competing risk)		
MDR vs. non-MDR	2.84 (1.05–7.69)	0.040
Charlson CCI, per 1 point	1.21 (1.04–1.41)	0.014
Recurrent UTI history	2.13 (0.91–4.99)	0.082

Hazard 1 in the Cox model indicates slower achievement of the outcome (clinical stability), i.e., delayed recovery. Schoenfeld residuals overall *p* = 0.41, supporting the proportional hazards assumption. Log-rank χ^2^ for MDR strata = 22.4, *p* < 0.001; median time to clinical stability 4.7 days (MDR) vs. 2.6 days (non-MDR). SHR, sub-distribution hazard ratio; TTE, time-to-effective therapy.

## Data Availability

The data presented in this study are available on request from the corresponding author.
